# Cardamonin suppressed the migration, invasion, epithelial mesenchymal transition (EMT) and lung metastasis of colorectal cancer cells by down-regulating ADRB2 expression

**DOI:** 10.1080/13880209.2022.2069823

**Published:** 2022-05-28

**Authors:** Ting Lu, Chunju Zheng, Zhimin Fan

**Affiliations:** a Proctology Department, Nanjing Hospital of Chinese Medicine Affiliated to Nanjing University of Chinese Medicine, Nanjing, China; b Proctology Department, Huai’an TCM Hospital Affiliated to Nanjing University of Chinese Medicine, Huai’an, China

**Keywords:** MMP-2, MMP-9, E-cadherin, N-cadherin, metastatic lung nodules

## Abstract

**Context:**

Cardamonin (CDN) can suppress cell growth in colorectal cancer (CRC), a common digestive malignancy.

**Objective:**

We explored the effect and mechanism of CDN on metastatic CRC.

**Materials and methods:**

Two cell lines (HT29 and HCT116) were initially treated with CDN at different concentrations (5, 10 and 20 μmol/L) or 50 μmol/L propranolol (positive control) for 24 or 48 h. Then, the two cell lines were separately transfected with siADRB2 and ADRB2 overexpression plasmids, and further treated with 10 μmol/L CDN for 24 h. The cell viability, migration and invasion were determined by cell counting kit-8 (CCK-8), wound healing and transwell assays, respectively. The levels of ADRB2, matrix metalloprotease (MMP)-2, MMP-9, E-cadherin and N-cadherin were measured by Western blotting or/and RT-qPCR. A CRC metastasis model was established to evaluate the antimetastatic potential of CDN (25 mg/kg).

**Results:**

ADRB2 (3.2-fold change; *p* < 0.001) was highly expressed in CRC tissues. CDN at 10 μmol/L suppressed viability (69% and 70%), migration (33% and 66%), invasion (43% and 72%) and ADRB2 expression (2.2- and 2.84-fold change) in HT29 and HCT116 cells (*p* < 0.001). CDN at 10 μmol/L inhibited MMP-2, MMP-9 and N-cadherin expression but promoted E-cadherin expression in CRC cells (*p* < 0.001). Importantly, the effect of CDN on CRC cells was impaired by ADRB2 overexpression, but further enhanced by ADRB2 down-regulation (*p* < 0.01). Additionally, ADRB2 overexpression reversed the inhibitory effect of CDN on metastatic lung nodules (*p* < 0.05). **Discussion and conclusions:** CDN is a potential candidate for the treatment of metastatic CRC in clinical practice.

## Introduction

Colorectal cancer (CRC) is one of the most common types of cancer that is responsible for numerous cancer-related deaths worldwide (Zhang Q et al. 2019). Based on global cancer statistics database, in 2018, about 1.09 million people suffered from CRC, of which 551,269 CRC patients died (Bray et al. 2018). The 5-year overall survival rate of patients with metastatic (stage IV) disease is 14% (James et al. 2017; Hou S et al. 2019), and metastatic disease occurs within 5 years in half of patients who underwent surgery for curative intent (Ferlay et al. 2015). Despite recent advances in the treatment of the disease, challenges still exist in the aspect of CRC metastasis which is extremely hard to be cured (Piawah and Venook 2019). Fortunately, targeted therapies, to our excitement, are now a part of the treatment modality for metastatic CRC, and may improve therapeutic outcome (Piawah and Venook 2019). Therefore, it is of great significance to discover molecular targeted therapeutic drugs for the treatment of metastatic CRC.

As a kind of pungent and warm Chinese herbal medicine, Caodoukou [also termed as semen Alpiniae katsumadai or *Alpinia katsumadai* Hayata (Zingiberaceae)] has various properties (Wang S et al. 2016). Cardamonin (2,4-dihydroxy-6-methoxychalcone, CDN), a natural chalcone compound, is the major active ingredient isolated from *Alpinia katsumadai* with potential effects in anti-inflammation and antitumor (Jin et al. 2019). For instance, CDN restrains the proliferation and metastasis of non-small-cell lung cancer cells (Zhou et al. 2019), and suppresses TGF-β1-provoked epithelial mesenchymal transition (EMT) of A549 cells (Kim et al. 2015). In addition, CDN inhibits proliferation, migration and invasion of prostate cancer cells (Zhang J et al. 2017), HT-1080 sarcoma cells (Park MK et al. 2013) and gastric cancer cells (Wang Z et al. 2019). These inhibitory effects of CDN on cancer metastasis make it a promising novel drug candidate to prevent or impede metastasis. A previous report demonstrated that CDN hinders the proliferation of CRC cells through enhancing β-catenin degradation (Park S et al. 2013). Moreover, a recent result verifies that CDN reduces chemotherapy resistance of CRC cells through the TSP50/NF-κB pathway *in vitro* (Lu et al. 2018). However, the precise effect and molecular mechanism of CDN on metastasis of CRC remain to be elucidated.

Metastatic dissemination represents the real reason of the malignant nature of tumours, the targeting of which is far more difficult than that of cell proliferation (Robert 2013). EMT is a critical metastasis-related process, where cancer cells gain the metastatic competence of migratory and invasive capabilities (Heerboth et al. 2015). Consequently, controlling EMT is fundamental to prevent and cure metastasis. In addition, accumulative evidence has indicated that adrenergic signalling plays an essential part in chronic stress-induced progression and metastasis in tumour (Zhang X et al. 2019). β_2_ Adrenergic receptor (ADRB2) specifically binds to and is also provoked by the endogenous class of ligands known as catecholamines and epinephrine (Litonjua et al. 2010). In cancer cells, ADRB2 modulates the proliferation, migration, apoptosis, angiogenesis and metastasis (Ha et al. 2019). ADRB2 is strikingly implicated with tumour grade, size, invasion and lymph node metastasis of CRC (Ciurea et al. 2017) and selective inhibition on ADRB2 can repress growth of CRC (Chin et al. 2016), indicating that ADRB2 might be a promising therapeutic target for combating the development of ADRB2-dependent CRC. The current research therefore aimed to explore the effect of CDN on biological behaviours mainly including EMT, migration and invasion of CRC cells, and further investigated whether these effects are associated with ADRB2 expression.

## Materials and methods

### Ethics statement

We collected 56 pairs of colorectal carcinoma tissues and corresponding normal mucosa tissues from CRC patients undergoing operation in the Nanjing Hospital of Chinese Medicine Affiliated to Nanjing University of Chinese Medicine from March 2018 to April 2020. The tissues were snap frozen in liquid nitrogen. The present study was reviewed and approved by the Ethics Committee of the Nanjing Hospital of Chinese Medicine Affiliated to Nanjing University of Chinese Medicine (ethical batch number: NJ201801005). All patients agreed to the use of their specimens before sampling.

This study was recommended by the Committee of Experimental Animals of Nanfang Hospital (ethical batch number: NF20200603015). All animal experiments were carried out in Nanfang Hospital complying with the principles of the China Council on Animal Care and Use. Every effort was made to minimize the pain and discomfort in the animals.

### Chemicals

Dimethyl sulphoxide (DMSO; D8371), BCA Protein Assay Kit (PC0020) and phosphate-buffered saline (PBS; P1010) powder with pH 7.3, cell counting kit-8 (CCK-8; CA1210), 4% paraformaldehyde (P1110), 1% crystal violet (C8470), RIPA buffer (R0010), ColorMixed Protein Marker (PR1910, 11–180 kDa) and ECL detection reagent (PE0010) were purchased from Solarbio (Beijing, China). Propranolol (Prop, P8688, an ADRB2 blocker) and DN (C16H14O4, purity ≥98%, C8249) were bought from Sigma-Aldrich (St. Louis, MO). Lipofectamine 3000 reagent (L3000008) was obtained from Thermo Fisher Scientific (Shanghai, China). Advantage RT-for-PCR Kit (639506) and TB Green^®^ Advantage^®^ qPCR Premix (639676) were acquired from TaKaRa (Dalian, China). Matrigel (354234) was ordered from BD Biosciences/Corning (Corning, NY). TRIzol (R0016) was available from Beyotime (Shanghai, China). Polybrene was procured from Gene Pharma (Shanghai, China).

### Cell culture

Human CRC cell lines HT29 (HTB-38) and HCT116 (CCL-247) as well as human normal colorectal fibroblasts CCD-18Co (CRL-1459) were purchased from American Type Culture Collection (ATCC, Manassas, VA). Cells were grown in Dulbecco's modified Eagle's medium (DMEM, 11995, Solarbio, Beijing, China) supplemented with 10% foetal bovine serum (FBS, C0232, Beyotime, Shanghai, China) and 1% penicillin–streptomycin (15070063, Gibco, Grand Island, NY) at 37 °C in a humidified atmosphere with 5% CO_2_.

### Transfection and treatment

ADRB2 overexpression vector was generated by inserting its full-length sequence (NCBI accession number: NC_000005.10) into the pUC57 vector purchased from GenePharma (Shanghai, China), with the empty pUC57 vector as a negative control (NC). SiADRB2 (siG000000154A-1-5) and siNC (siN0000002-1-5) were ordered from RiboBio Co. Ltd. (Guangzhou, China). SiNC served as the control for siADRB2. HT29 or HCT116 cells were seeded in a six-well plate at a density of 3.0 × 10^5^ cells/well. When cell confluence reached 70%, ADRB2 overexpression vector, empty pUC57, siADRB2 and siNC were separately transfected into cells using Lipofectamine 3000 reagent for 24 h. CDN was dissolved in DMSO. HT29 and HCT116 cells were treated with CDN at various concentrations (5, 10 and 20 μmol/L) for 24 or 48 h, and with 50 μmol/L Prop for 24 h (Wang F et al. 2018) as a positive control. To further explore the mechanism of CDN in CRC, the expression of ADRB2 was up-regulated or down-regulated using ADRB2 overexpression vector or siADRB2 in HT29 and HCT116 cells that were then treated with CDN. Controls were only exposed to culture media containing 0.5% (v/v) DMSO.

### Cell viability

The viability of HT29 and HCT116 cells was detected by CCK-8. In short, HT29 and HCT116 cells with or without transfection were plated in a 96-well plate at a density of 1.0 × 10^4^ cells/well and incubated overnight. Subsequently, the cells were treated with CDN at different concentrations. After culture for indicated times, CCK-8 solution (10 μL) was added to each well for additional 1 h of incubation. Next, cell viability was assessed by detecting absorbance at a wavelength of 450 nm with a Microplate Reader (GENios-Pro, Tecan, Milan, Italy).

### Wound healing assay

To reveal the migration capacity, the wound healing assay was performed as previously reported (Gu et al. 2019). The transfected or untransfected HT29 and HCT116 cells were separately placed into six-well plates (3 × 10^5^ cells/well). When the cells reached about 90% confluence, a 200 μL pipette tip was used to make separate wounds on cell monolayers. Afterwards, the damaged cells were washed with PBS, and the adherent cells were cultured in FBS-free medium with CDN or Prop at the indicated concentrations. The distance covered by migrated cells was quantified at 0 and 24 h utilizing a microscope (×100 magnification, E800, Nikon, Tokyo, Japan).

### Transwell assay

To uncover cell invasion capacity, the transwell assay was carried out in accordance with a previous report (Gu et al. 2019). The 24-well chambers (#3422, 8 μm pore size, Corning, Corning, NY) were pre-coated with 50 mg/L Matrigel (1:8). After transfection, HT29 and HCT116 cells were starved in FBS-free medium for 24 h. Subsequently, 200 μL cells were added into the upper chamber (3 × 10^5^ cells/well) and 500 μL medium containing 20% FBS with CDN or Prop at the indicated concentrations was transferred to the lower chamber. After incubation at 37 °C for 24 h, the invading cells were fixed with 4% paraformaldehyde for 10 min and then dyed with 1% crystal violet for 30 min. The invasion ability of cells was determined with a microscope (×100 magnification).

### Reverse transcription-quantitative polymerase chain reaction (RT-qPCR)

Total RNA was isolated from cells and tissues with TRIzol and reverse-transcribed into complementary DNAs (cDNAs) with Advantage RT-for-PCR Kit. The cDNAs were amplified based on the standard qPCR protocol with TB Green^®^ Advantage^®^ qPCR Premix in an Applied Biosystems 7500 Fast Real-Time PCR System (Foster City, CA). Quantitative PCR was conducted at 95 °C for 10 min, followed by 35 cycles of 95 °C for 15 s and 60 °C for 60 s. Primers used in this research are listed in Table 1. GAPDH acted as the internal control and the results were calculated using 2^–ΔΔCt^ method (Livak and Schmittgen 2001).

**Table 1.** Primer sequences used for quantitative reverse transcription polymerase chain reaction (qRT-PCR) of human genes.

Genes	Primer sequences (5′–3′)
ADRB2	
Forward	TTGCTGGCACCCAATAGAAGC
Reverse	CAGACGCTCGAACTTGGCA
MMP-2	
Forward	TACAGGATCATTGGCTACACACC
Reverse	GGTCACATCGCTCCAGACT
MMP-9	
Forward	TGTACCGCTATGGTTACACTCG
Reverse	GGCAGGGACAGTTGCTTCT
E-cadherin	
Forward	GTT ATT CCT CTC CCA TCA GCT G
Reverse	CTT GGC TGA GAG GAT GGT GTA A
N-cadherin	
Forward	TCAGGCGTCTGTAGAGGCTT
Reverse	ATGCACATCCTTCGATAAGACTG
GAPDH	
Forward	AGCCACATCGCTCAGACAC
Reverse	GCCCAATACGACCAAATCC

### Western blotting

Western blotting was performed as previously reported (Gu et al. 2019). Whole-cell lysates were isolated from cells and tissues with RIPA buffer. Protein concentration was determined with BCA Protein Assay Kit. ColorMixed Protein Marker was applied as a protein size marker. The protein lysate (25 µg) was then separated by 6–10% sodium dodecyl sulphate-polyacrylamide gel electrophoresis (SDS-PAGE) and transferred to a PVDF membrane (3010040001, Sigma-Aldrich, St. Louis, MO). After being blocked with 5% non-fat milk at 37 °C for 1 h, the membrane was incubated first with primary antibodies at 4 °C overnight and then with corresponding secondary antibodies at 37 °C for 1 h. The protein was visualized using an ECL detection reagent and quantified by means of Bio-Rad ChemiDoc system (version 6.0 Bio-Rad Laboratories, Inc., Hercules, CA) with software Image J v2.1.4.7 (National Institutes of Health, Bethesda, MD). Antibodies used are listed in Table 2. GAPDH served as the internal control.

**Table 2.** List of primary antibodies used for Western blots.

Protein	Antibody	Catalogue number	Company	Antibody dilution
MMP-2	Rabbit anti-MMP2 antibody	ab37150	Abcam	1:200
MMP-9	Rabbit anti-MMP9 antibody	ab38898	Abcam	1:1000
E-cadherin	Mouse anti-E cadherin	ab1416	Abcam	1:50
N-cadherin	Rabbit anti-N cadherin	ab18203	Abcam	1:1000
GAPDH	Rabbit anti-GAPDH	ab181602	Abcam	1:10,000
Secondary anti-body	Goat anti-rabbit IgG H&L (HRP)	ab205718	Abcam	1:2000
Secondary anti-body	Goat anti-mouse IgG H&L (HRP)	ab205719	Abcam	1:2000

### Animal studies

Six-week-old female BALB/c nude mice purchased from Shanghai Animal Laboratory Centre (Shanghai, China) were used to conduct all the *in vivo* studies. The mice were reared in Laboratory Animal Centre of Nanfang Hospital in a specific pathogen-free atmosphere.

For the metastasis model (Zhu et al. 2020), mice were randomly divided into five groups (*n* = 3) as follows: normal, control, CDN, NC + CDN and ADRB2 + CDN groups.

- *Normal group*: Mice were injected with 200 µL PBS through the tail veins;
- *Control group*: Mice were injected with 200 µL HT29 cell suspension (1 × 10^7^ cells/mL) through the tail veins;
- *CDN group*: Mice were injected with 200 µL HT29 cell suspension (1 × 10^7^ cells/mL) through the tail veins. Seven days later, mice were treated with CDN (25 mg/kg) (Hou G et al. 2020) twice a week via intraperitoneal injection;
- *NC + CDN group*: Mice were injected through the tail veins with 200 µL HT29 cell suspension (1 × 10^7^ cells/mL) that was infected with lentivirus control by 8 mg/mL polybrene. After seven days, mice were treated with CDN (25 mg/kg) twice a week via intraperitoneal injection;
- *ADRB2 + CDN group*: Mice were injected through the tail veins with 200 µL HT29 cell suspension (1 × 10^7^ cells/mL) that was infected with ADRB2 sequence by 8 mg/mL polybrene. Seven days later, mice were treated with CDN (25 mg/kg) twice a week via intraperitoneal injection.

After 28 days of continuous treatment, the mice were sacrificed. The number of metastatic lung nodules was counted.

### Data analysis

**Figure 1.**
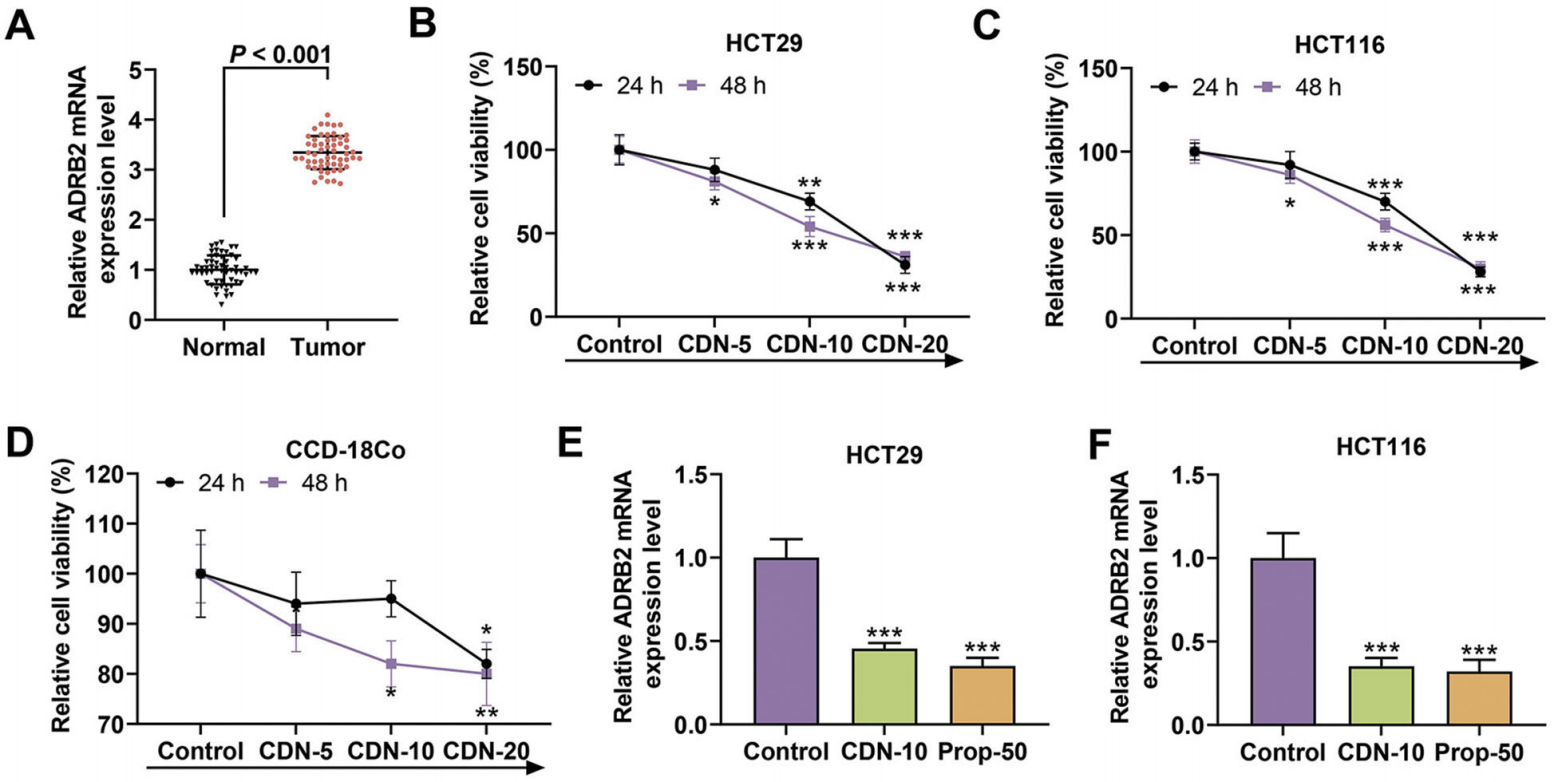
CDN suppressed viability and ADRB2 expression in CRC cells. (A) The mRNA level of ADRB2 in human CRC tissues and matched normal tissues was determined by RT-qPCR. (B–D) The viability of HT29, HCT116 and CCD-18Co cells treated with CDN at various concentrations (5, 10 and 20 μmol/L) for 24 or 48 h was examined by CCK-8. (E, F) The ADRB2 mRNA level in HT29 and HCT116 cells treated with CDN at 10 μmol/L was examined by RT-qPCR. *p < 0.5 or ***p < 0.001 vs. control. GAPDH acted as the internal control. Control: cells only exposed to culture media containing 0.5% (v/v) DMSO; CDN: cardamonin; CRC: colorectal cancer; ADRB2: β_2_ adrenergic receptor; Prop-50: propranolol (50 μmol/L); CCK-8: cell counting kit-8; DMSO: dimethyl sulphoxide; RT-qPCR: reverse transcription-quantitative polymerase chain reaction.

The experiment was repeated three times. GraphPad prism 8.0 software (GraphPad Software, San Diego, CA) was utilized for processing the data that were presented as mean ± standard deviation (SD). A paired *t*-test was used to analyse paired samples (Figure 1(A)). Comparison between two groups was fulfilled using Student’s *t*-test, and comparison among multiple groups was achieved by analysis of variance (ANOVA) followed by *post hoc* Tamhane’s test. *p* < 0.05 was considered as statistically significant.

## Results

### CDN suppressed CRC cell viability and expression of ADRB2 which was high-expressed in CRC

As shown in Figure 1(A), the mRNA level of ADRB2 was higher in CRC tissues than in normal tissues (*p* < 0.001). In addition, CCK-8 results indicated that CDN concentration-dependently restrained the viability of HT29 and HCT116 cells at 24 and 48 h (Figure 1(B,C), *p* < 0.05). When the concentration of CDN exceeded 10 μmol/L at 24 and 48 h, the viability of HT29 and HCT116 cells was less than 50%, but that of CCD-18Co cells was greater than 50% (Figure 1(D)). Thus, CDN at a concentration of 10 μmol/L was chosen for all subsequent experiments. It can be noted from RT-qPCR results that the mRNA level of ADRB2 in HT29 and HCT116 cells was strikingly suppressed by CDN and Prop (Figure 1(E,F), *p* < 0.001).

### CDN hindered migration and invasion of CRC cells

**Figure 2.**
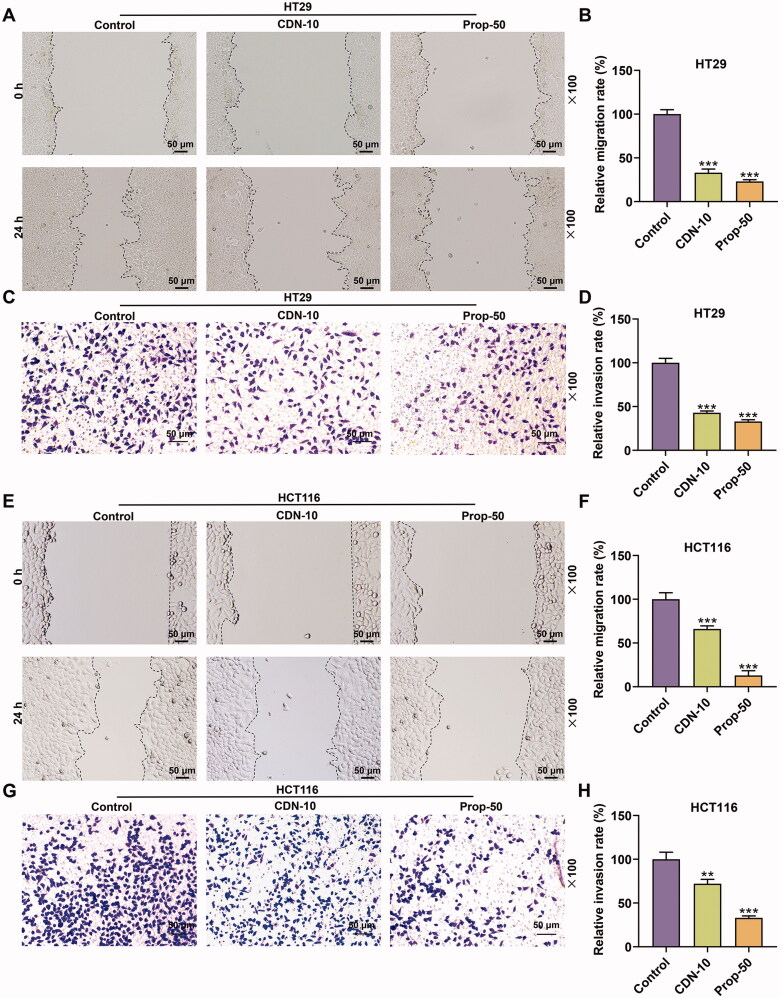
CDN inhibited migration and invasion of CRC cells. The migration and invasion of HT29 (A–D) and HCT116 cells (E–H) treated with CDN at 10 μmol/L were examined using wound healing and transwell assays, respectively. **p < 0.01 or ***p < 0.001 vs. control. Control: cells only exposed to culture media containing 0.5% (v/v) DMSO; DMSO: dimethyl sulphoxide; CDN: cardamonin; CRC: colorectal cancer; Prop-50: propranolol (50 μmol/L).

According to Figure 2(A), in control group, cell wound width was obviously reduced at 24 h when compared to that at 0 h. By contrast, in CDN group or Prop-50 group, the wound width at 24 h showed no obvious changes compared to that at 0 h.

Figure 2(B) depicts that the migration rate of HT29 cells in CDN or Prop-50 group was evidently decreased (*p* < 0.001) in contrast with that in control group. Also, similar results were observed in HCT116 cells (Figure 2(E–H), *p* < 0.001).

### CDN inhibited the expression of matrix metalloprotease (MMP)-2, MMP-9 and N-cadherin, but promoted E-cadherin expression in CRC cells

**Figure 3.**
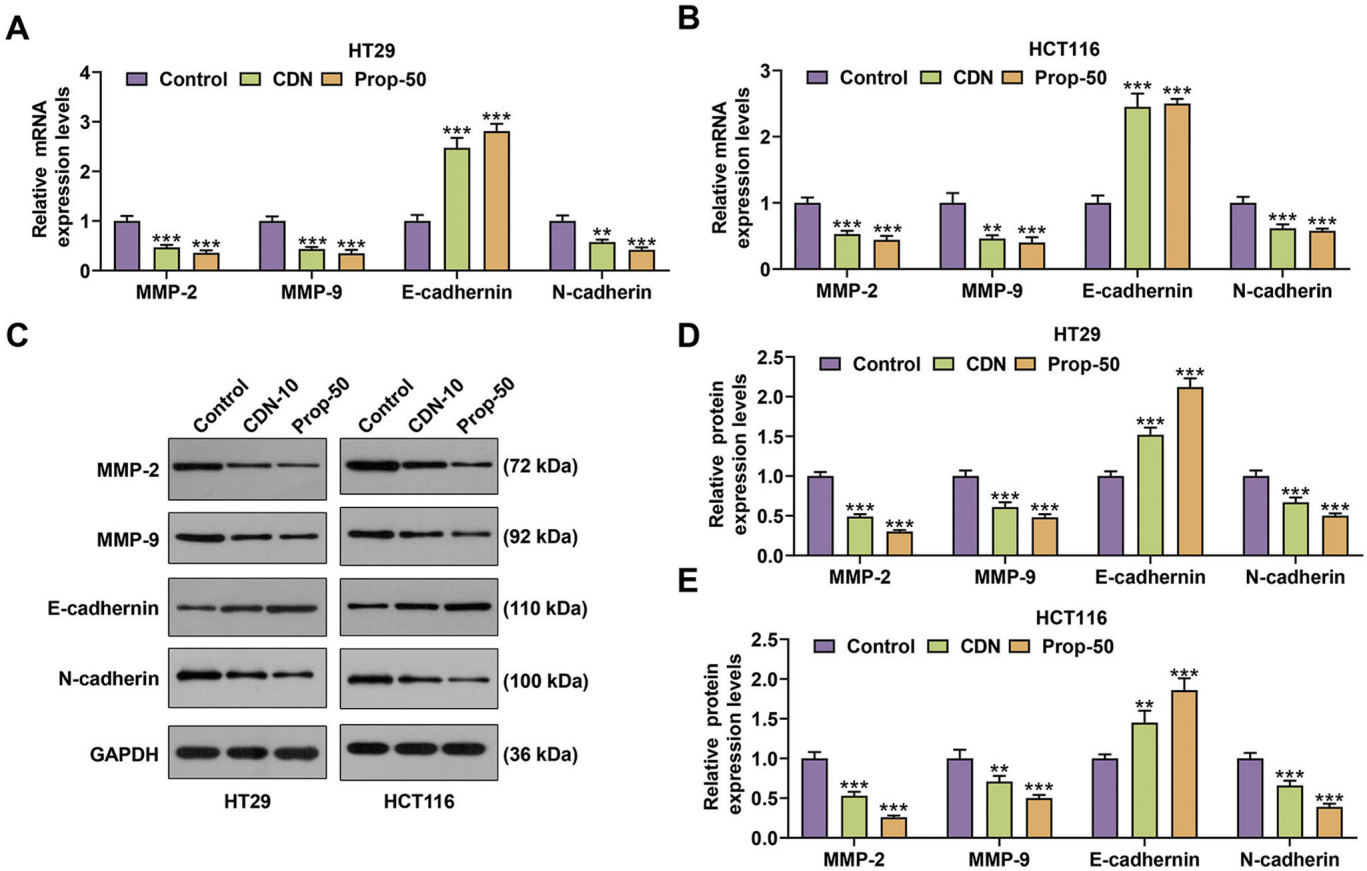
CDN decreased the expression of MMP-2, MMP-9 and N-cadherin, but increased E-cadherin expression in CRC cells. (A–F) The mRNA and protein levels of MMP-2, MMP-9, E-cadherin and N-cadherin in HT29 (A–C) and HCT116 (D–F) cells treated with CDN at 10 μmol/L were examined using RT-qPCR and Western blotting. **p < 0.01 or ***p < 0.001 vs. control. GAPDH acted as the internal control. Control: cells only exposed to culture media containing 0.5% (v/v) DMSO; DMSO: dimethyl sulphoxide; CDN: cardamonin; Prop-50: propranolol (50 μmol/L); CRC: colorectal cancer; RT-qPCR: reverse transcription-quantitative polymerase chain reaction; MMP: matrix metalloprotease.

RT-qPCR and Western blotting data demonstrated that the expression of MMP-2, MMP-9 and N-cadherin at mRNA and protein levels was markedly inhibited by CDN in HT29 (Figure 3(A–C), *p* < 0.001) and HCT116 (Figure 3(D–F), *p* < 0.001) cells, whilst that of E-cadherin was prominently enhanced (Figure 3(A–F), *p* < 0.01). Similar results were also shown in cells treated with 50 μmol/L Prop (Figure 3(A–E), *p* < 0.001).

### CDN attenuated viability of CRC cells through inhibiting ADRB2 expression

**Figure 4.**
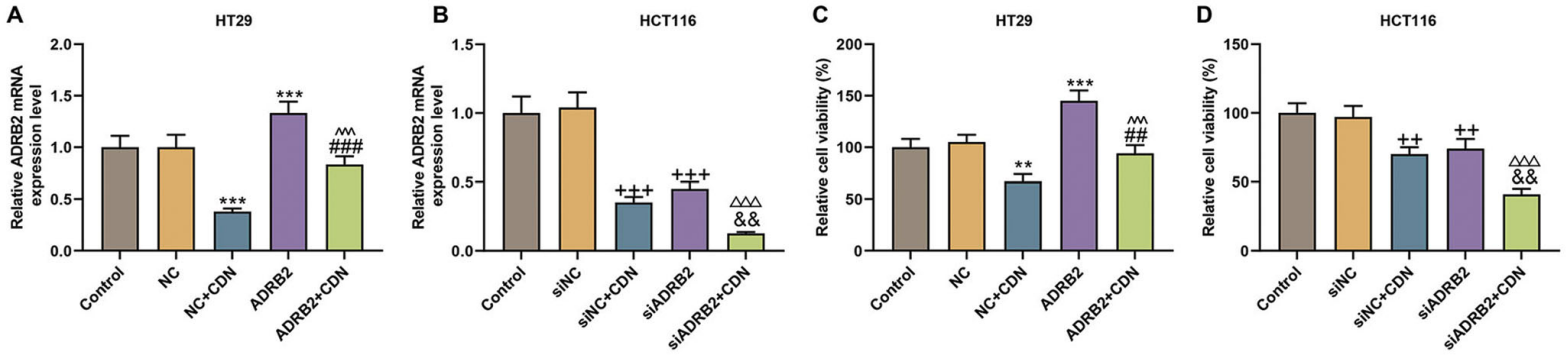
CDN suppressed ADRB2 expression and viability of CRC cells through inhibiting ADRB2 expression. Cells transfected with siADRB2 or ADRB2 overexpression plasmid were exposed to CDN and then examined for the mRNA level of ADRB2 and the viability using RT-qPCR (A, B) and CCK-8 (C, D), respectively. GAPDH acted as the internal control. **p < 0.01 or ***p < 0.001 vs. NC; ^##^*p* < 0.01 or ^###^*p* < 0.001 vs. NC + CDN; ^^^^^*p* < 0.001 vs. ADRB2; ^++^*p* < 0.01 or ^+++^*p* < 0.001 vs. siNC; ^&&^*p* < 0.01 vs. NC + CDN-10; ^△△△^*p* < 0.001 vs. siADRB2. NC: negative control (empty vector); siNC: negative control of small interfering RNA (siRNA); CDN: cardamonin; CRC: colorectal cancer; ADRB2: β_2_ adrenergic receptor; CCK-8: cell counting kit-8; RT-qPCR: reverse transcription-quantitative polymerase chain reaction; siADRB2: siRNA for ADRB2.

ADRB2 expression (Figure 4(A), *p* < 0.001) and viability (Figure 4(C), *p* < 0.001) were notably promoted by ADRB2 overexpression in HT29 cells, but were remarkably repressed by ADRB2 knockdown (Figure 4(B), *p* < 0.001; Figure 4(D), *p* < 0.01) in HCT116 cells. Moreover, the inhibitory effects of CDN on ADRB2 expression and viability were effectively reversed by ADRB2 overexpression in HT29 cells (Figure 4(A,C), *p* < 0.01), but were dramatically promoted by ADRB2 knockdown in HCT116 cells (Figure 4(B,D), *p* < 0.01).

### CDN impeded migration and invasion of CRC cells through inhibiting ADRB2 expression

**Figure 5.**
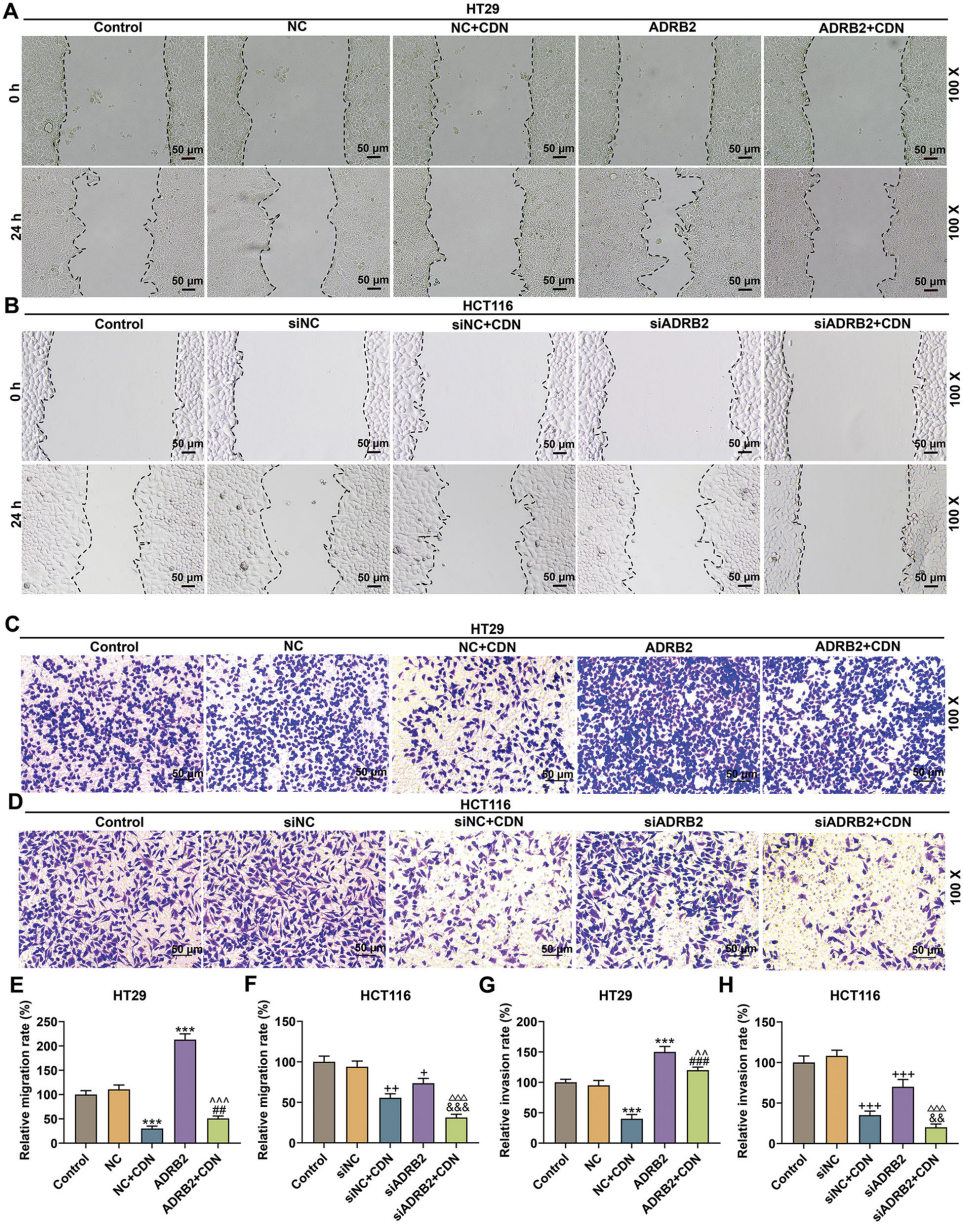
CDN blocked migration and invasion of CRC cells through inhibiting ADRB2 expression. Cells transfected with siADRB2 or ADRB2 overexpression plasmid were exposed to CDN and then examined for the migration and the invasion by wound healing assay (A, B, E and F) and transwell assay (C, D, G and H), respectively. ***p < 0.001 vs. NC; ^##^*p* < 0.01 or ^###^*p* < 0.001 vs. NC + CDN; ^^^^*p* < 0.05 or ^^^^^*p* < 0.001 vs. ADRB2; ^+^*p* < 0.05 or ^++^*p* < 0.01 or ^+++^*p* < 0.001 vs. siNC; ^&&&^*p* < 0.001 vs. NC + CDN-10; ^△△△^*p* < 0.001 vs. siADRB2. NC: negative control (empty vector); siNC: negative control of small interfering RNA (siRNA); CDN: cardamonin; CRC: colorectal cancer; ADRB2: β_2_ adrenergic receptor; siADRB2: small interfering RNA for ADRB2.

The migration (Figure 5(A,E), *p* < 0.001) and invasion (Figure 5(C,G), *p* < 0.001) of HT29 cells were obviously boosted by ADRB2 overexpression, but those (Figure 5(B,F), *p* < 0.001; Figure 5(D,H), *p* < 0.001) in HCT116 cells were markedly repressed by ADRB2 knockdown. Furthermore, the inhibitory effects of CDN on migration and invasion of HT29 cells (Figure 5(A,C,E,G), *p* < 0.01) were notably offset by ADRB2 overexpression, but those on migration and invasion of HCT116 cells (Figure 5(B,F,D,H), *p* < 0.01) were observably promoted by ADRB2 knockdown.

### CDN down-regulated MMP-2, MMP-9 and N-cadherin expression but up-regulated E-cadherin expression in CRC cells through suppressing ADRB2 expression

**Figure 6.**
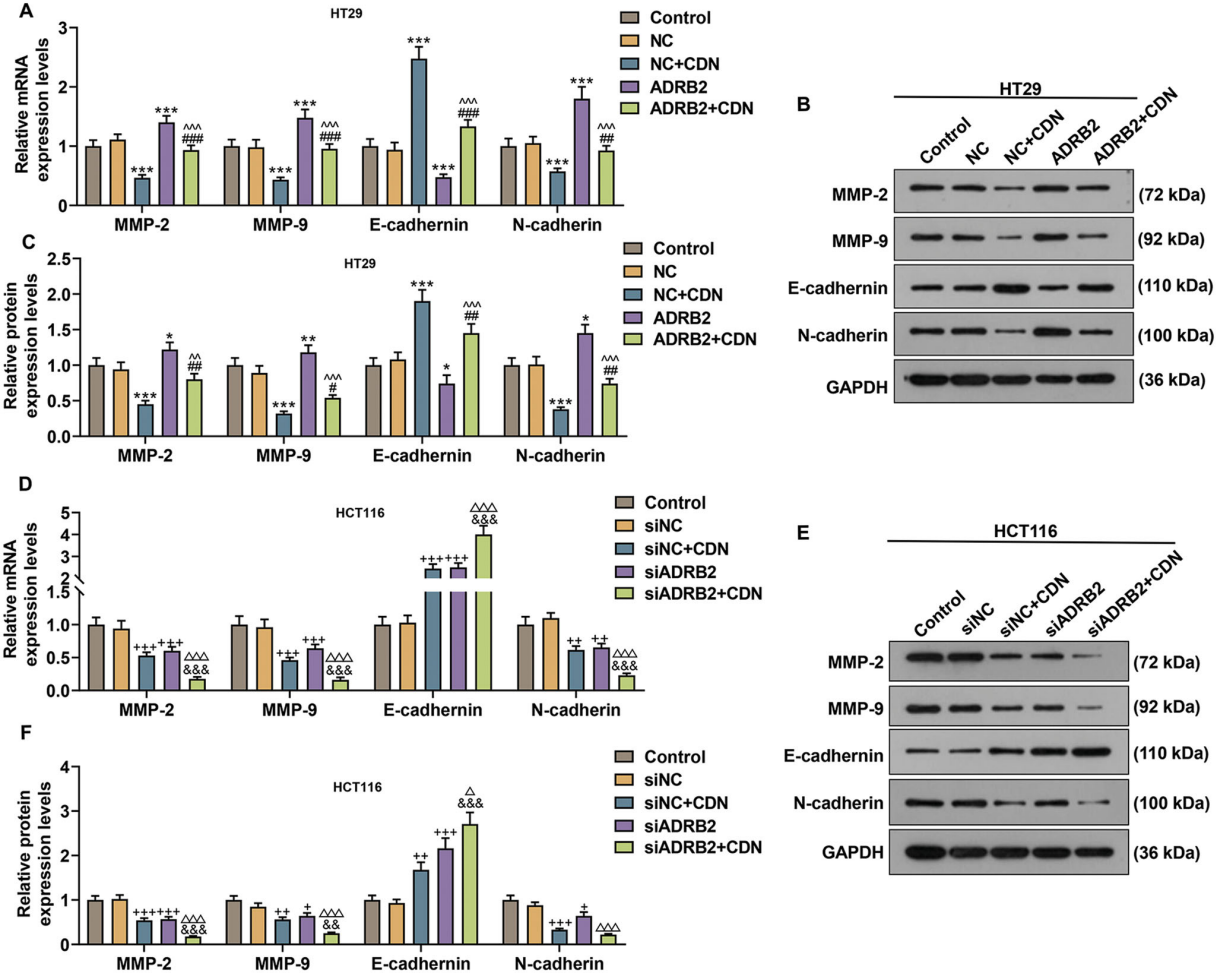
CDN lessened MMP-2, MMP-9 and N-cadherin expression but augmented E-cadherin expression in CRC cells through inhibiting ADRB2 expression. (A–F) ADRB2 overexpression or knockdown was achieved by transfection of ADRB2 overexpression plasmid or siADRB2 into CRC cells, respectively. Transfected cells were exposed to CDN and then examined for the mRNA and protein levels of MMP-2, MMP-9, E-cadherin and N-cadherin using RT-qPCR (A, D) and Western blotting (B, C, E and F). GAPDH acted as the internal control. ***p < 0.001 vs. NC; ^##^*p* < 0.01 or ^###^*p* < 0.001 vs. NC + CDN; ^^^^*p* < 0.05 or ^^^^^*p* < 0.001 vs. ADRB2; ^+^*p* < 0.05 or ^++^*p* < 0.01 or ^+++^*p* < 0.001 vs. siNC; ^&&^*p* < 0.05 or ^&&&^*p* < 0.001 vs. NC + CDN-10; ^△△△^*p* < 0.001 vs. siADRB2. NC: negative control (empty vector); siNC: negative control of small interfering RNA (siRNA); CDN: cardamonin; CRC: colorectal cancer; ADRB2: β_2_ adrenergic receptor; RT-qPCR: reverse transcription-quantitative polymerase chain reaction; MMP: matrix metalloprotease; siADRB2: small interfering RNA for ADRB2.

MMP-2, MMP-9 and N-cadherin expression was remarkably elevated but E-cadherin expression was dwindled in HT29 cells transfected with ADRB2 overexpression vector (Figure 6(A–C), *p* < 0.05), whilst the opposite changing trends were noticed in HCT116 cells transfected with ADRB2 knockdown (Figure 6(D,E), *p* < 0.05). Additionally, the effects of CDN on MMP-2, MMP-9, E-cadherin and N-cadherin expression was effectively reversed by ADRB2 overexpression in HT29 cells (Figure 6(A–C), *p* < 0.001), but were reinforced by ADRB2 knockdown in HCT116 cells (Figure 6(D,E), *p* < 0.05).

### ADRB2 overexpression reversed the inhibitory effect of CDN on metastatic lung nodules in CRC metastasis model

**Figure 7.**
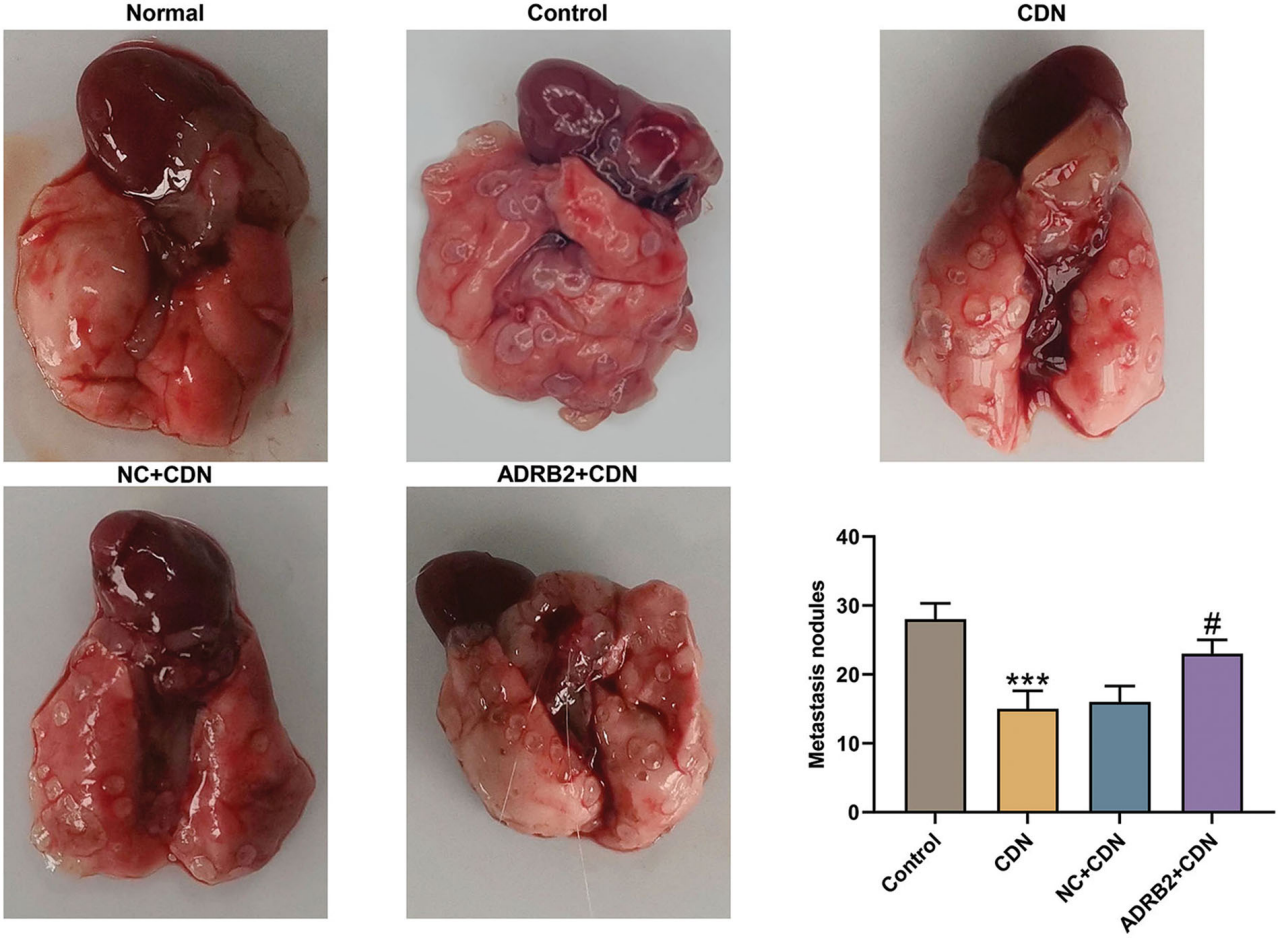
ADRB2 overexpression reversed the inhibitory effect of CDN on metastatic lung nodules in CRC metastasis model. (A) In the presence of pulmonary metastatic nodules in nude mice 5 weeks after tail vein injection of the tumour cells, the number of metastatic nodules in the lung was counted. ***p < 0.001 vs. control; ^#^*p* < 0.05 vs. NC + CDN. CDN: cardamonin; CRC: colorectal cancer.

As delineated in Figure 7, the metastatic lung nodules were lessened in CDN group compared to those in control group (*p* < 0.001), whereas the effect of CDN was partially reversed by ADRB2 overexpression (*p* < 0.05).

## Discussion

To the best of our knowledge, this is the first study to elucidate the molecular mechanism underlying the inhibitory effect of CDN on lung metastasis of CRC via regulation of ADRB2 expression. The present study revealed that CDN inhibited ADRB2 expression, viability, migration, invasion and EMT of CRC cell lines (HT29 and HCT116) via down-regulating ADRB2 expression. Furthermore, a metastasis mouse model was used to confirm the findings *in vitro*, which emphasized the importance of CDN as a promising therapy to lung metastasis in CRC patients.

To initiate metastasis, cancer cells undergo a phenotypic alteration from epithelial cells to mesenchymal cells, a process known as EMT (Hassan et al. 2017). EMT is a possible mechanism of migration, invasion and subsequent metastasis in cancers (Xu et al. 2020). In certain tumour types, the critical markers of EMT are loss of E-cadherin (epithelium-derived labelled protein) expression and induction of N-cadherin (the labelled protein of the mesenchymal cells) expression (Molnár et al. 2019). In the EMT process, mesenchymal-like pro-migratory phenotypes enable cells to cross anatomical boundaries such as the extracellular matrix (ECM) or the basement membrane (Kryczka et al. 2019). Additionally, MMP-2 and MMP-9 are responsible for ECM degradation and basement membrane impairment (Angelone et al. 2017). In this study, CDN suppressed viability of CRC cells, which was consistent with Park’s findings (Park S et al. 2013). Moreover, previous studies also put forward that CDN retards migration, invasion and EMT of cancer cells (Park MK et al. 2013; Shrivastava et al. 2017; Gmerek et al. 2019; Zhou et al. 2019; Zhang L et al. 2021). Our novel data extended previous observations and provided the first evidence to directly demonstrate that CDN suppressed cell migration, invasion and EMT through upregulation of E-cadherin and downregulation of N-cadherin, and facilitated ECM degradation via down-regulations of MMP-2 and MMP-9 in CRC cells. Therefore, CDN may serve as a therapeutic drug in treating metastatic CRC.

However, the targeted molecular mechanism of CDN on the treatment of metastatic CRC remains to be explored. It has been reported that ADRB2 expression is up-regulated in gastric cancer (Zhang X et al. 2019) and tongue squamous cell carcinoma (Liu et al. 2018). The current data implicated that ADRB2 was over-expressed in CRC tissues but was strikingly under-expressed in CRC cells treated with CDN, indicating that ADRB2 may act as an oncogene involved in the regulation of CDN on CRC progression. To further explore whether ADRB2 mediates the function of CDN in metastasis of CRC, the expression of ADRB2 was manipulated in HT29 and HCT116 cells by transfection with siADRB2 or ADRB2 overexpression plasmid. Previous studies corroborated that ADRB2 activation inhibits tumour progression (migration, invasion, EMT or metastasis) of oral cancer cells (Chen et al. 2019), breast cancer cells (Massaro et al. 2020) and tongue squamous cell carcinoma cells (Liu et al. 2018). The present study uncovered that ADRB2 overexpression strikingly promoted viability, migration and invasion and remarkably up-regulated MMP-2, MMP-9 and N-cadherin expressions, but suppressed E-cadherin expression in HT29 cells. By contrast, ADRB2 deficiency produced the opposite effects on the same aspects in HCT116 cells. Furthermore, ADRB2 overexpression reversed the inhibitory effect of CDN on metastasis nodules of lung in mouse metastasis model. The data verified that CDN inhibited migration, invasion and EMT of CRC cells as well as lung metastasis through targeting ADRB2, manifesting that CDN may suppress metastasis of CRC as a molecular targeted therapeutic drug.

Recent publications have reported that CDN attenuates CRC cell growth through signal transducers and transcription (STAT) signal activators (Hou S et al. 2019). CDN inhibits tumorigenesis in CRC through regulation of microRNAs (miRNAs) expression (James et al. 2017, 2021). Increasing evidence has indicated that miRNAs play important roles in the development and treatment of CRC (Chen et al. 2019; Gmerek et al. 2019; Massaro et al. 2020; Wang H 2020). Other reports have also demonstrated the importance of STAT signal in regulation of CRC development (Jiang et al. 2019; Fang et al. 2020; Dariya et al. 2021). Thus, there is a possibility for the potential crosstalk between ADRB2 and STAT3 or miRNA in CRC. This would be further explored in our future research.

Several limitations existed in this study that should be overcome in the future study. First, several experiments were performed without a positive control, which therefore should be further investigated in the future study. Second, more extensive investigations in clinical trials are required to further confirm our results.

## Conclusions

The present research demonstrates that CDN could suppress the CRC cell viability, migration and invasion *in vitro* and the metastasis to lung *in vivo*. The potential mechanism attributes to CDN-induced down-regulation of ADRB2, which further increases the level of E-cadherin, decreases those of N-cadherin, MMP-2 and MMP-9, and finally prevents EMT. These findings indicate that CDN may be perceived as a molecular targeted therapeutic drug for metastatic CRC.
